# It’s Time to Finally Kill the Zombies Comment on "Universal Pharmacare in Canada"

**DOI:** 10.15171/ijhpm.2020.03

**Published:** 2020-01-12

**Authors:** Joel Lexchin

**Affiliations:** ^1^School of Health Policy and Management, Faculty of Health, York University, Toronto, ON, Canada.; ^2^University Health Network, Toronto, ON, Canada.; ^3^Department of Family and Community Medicine, Faculty of Medicine, University of Toronto, Toronto, ON, Canada.

**Keywords:** Canada, Drug Expenditures, Formulary, Pharmacare, Pharmaceutical Industry

## Abstract

The movement for a national pharmacare plan in Canada is growing, but at the same time the multinational pharmaceutical companies and their supporters are critical of such a move. The three major arguments that they make are that all that is needed is to "fill in the gaps," ie, cover those who currently are uninsured or underinsured, that private drug plans are superior to public ones because they cover a larger number of drugs and that Canada cannot afford pharmacare. This commentary examines each of these arguments and makes the case that none of them is valid and that it is time to get on with implementing pharmacare.

Two recent articles in the *International Journal of Health Policy and Management* have separately made the case why Canada should adopt a universal, public pharmacare plan. Hajizadeh and Edmonds^1^ use data from six nationally representative surveys of Household Spending conducted by Statistics Canada between 2010 and 2015 to show how inequitable the coverage is for medicines when it comes to out-of-pocket payments. Depending on where you live in Canada as few as 1 in 20 households to as many as 1 in 10 households are spending 6% of their income on prescription drugs. Lewis^2^ runs through the key players in the pharmacare debate – physicians, chain pharmacies, private drug insurers, the pharmaceutical industry and the public – and discusses how each will advance or impede the movement towards pharmacare.

The prospects for pharmacare, a plan that would ensure access to all medically necessary drugs for Canadians at minimal to no out-of-pocket cost, are better now than they have been since the early 1970s.^3^ The mandate letter from Prime Minister Justin Trudeau to his Minister of Health calls for her to implement “national universal pharmacare”^4^ and the social democratic New Democratic Party, the most likely ally of the Liberals in the new minority government, has placed pharmacare at the top of its priority list. However, the going will not be all smooth. The mandate letter is vague about what any future pharmacare plan will look like and the provincial and territorial premiers are, at best, lukewarm to the idea. Their first priority for the federal government is for it to increase its transfers for current healthcare services by 5.2% annually. After that, they may be willing to consider pharmacare if provinces have an opt out clause.^5^

But even before pharmacare has a chance to be born, the knives are out to try and ensure that it never sees the light of day. Who is wielding those knives? The answer to that question comes from an essay by one of Canada’s pre-eminent health economists, Robert Evans. Evans and colleagues^6^ remind us that “Every dollar of expenditure is a dollar of someone’s income.” The income earners in this case are the pharmaceutical companies and to a lesser extent the private insurance companies.

Pharmacare means monopsony buying power for the government and the ability to extract large discounts. For example, Australia, through its Pharmaceutical Benefits Scheme, struck a 5-year deal with pharmaceutical companies to supply the country with all of the antiretroviral drugs necessary to treat the 230 000 Australians with hepatitis C for A$1 billion, thereby lowering the per person treatment cost from A$20 000 to about A$4300.^7^ There is also the inevitable reality that not all drugs will be listed on a national formulary since only about 1 in 10 new drugs are significantly therapeutically superior to already existing medicines.^8^

So it should not be any surprise that the knives are in the hands of the brand-name pharmaceutical industry,^9^ its supporters^10-12^ and the insurance companies.^13^ They all agree that all Canadians should be covered but they insist that national pharmacare is not the way to go. The arguments vary somewhat, but typically emphasize three themes: most Canadians are insured so we only need to fill in the gaps, pharmacare will deprive people of drugs that their private insurance is paying for and pharmacare is too costly and we cannot afford it. None of those arguments bears scrutiny.

About 60% of Canadians are covered by private insurance and many of the rest qualify for one of the provincial public insurance plans, but that still leaves almost 1 in 10 Canadians with cost-related nonadherence, especially those who are poor and/or in poor health.^14^ The province of Quebec tried to deal with this problem by requiring any employer who offers health benefits to include drugs in those benefits and for everyone else the government steps in. On some measures such as cost-related nonadherence Quebec does better than other provinces but given the poor coverage in other provinces that is not the right comparison. The right comparison is with countries that provide universal coverage and on that measure Quebec lags behind countries such as Australia, Germany, the Netherlands and the United Kingdom. Similarly, a greater percentage of people in Quebec report spending more than $1000 out-of-pocket on drugs and total per capita spending on drugs in Quebec ($1087) is substantially higher than the average in the rest of Canada ($912) and countries with universal coverage ($826).^15^ Moreover, continuing to heavily rely on private insurance means higher administrative costs, less generic substitution, annual or lifetime caps on coverage for some people and taxpayer subsidies since health benefits are provided tax free.^16-19^

Defenders of the current system make much of the fact that private insurance plans cover a greater percent of drugs approved by Health Canada than do public provincial plans. Citing research by the Canadian Health Policy Institute, the Canadian Life and Health Insurance Association makes the case that private insurance is superior to public insurance because of the 479 new drugs approved by Health Canada from 2008 to 2017, 87% were covered by at least one private drug plan, compared to 45% that were covered by at least one public plan.^20^ Similarly, a report from Innovative Medicines Canada, the lobby group for the multinational drug companies operating in Canada, claims that Canada ranked 18th out of 20 Organisation for Economic Co-operation and Development (OECD) countries in terms of the percent of new drugs and new drug combinations introduced between January 1, 2010 and December 31, 2014 that were publicly funded.^21^ (I made three attempts to contact Innovative Medicines Canada to obtain the list of drugs that were evaluated in this report but never received an answer.)

The rationale behind the notion that the more drugs covered the better the plan, is based on work by Frank Lichtenberg who claims that more use of newer medicines is associated with lower morbidity, mortality and overall healthcare spending.^22^ However, Lichtenberg’s conclusions are heavily contested by a number of authors.^23-26^ (Lichtenberg has responded to his critics and defended his work.^27,28^) Nor do more drugs equate to more consumer choice, even ignoring that it is doctors who actually make the choice. More than half of the new drugs and new indications for existing drugs introduced onto the French market were rated as “nothing new” by the independent French drug bulletin *Prescrire International* and 17% were judged to have a negative benefit to harm ratio and should never have been approved.^29^

As of 2013, most private drug plans were not using formularies,^30^ but that situation is changing and in recent years two large plans announced the use of formularies.^31,32^ If a national formulary for pharmacare is “well-constructed by disinterested experts” as Lewis recommends, with a “safety valve of a separate pool to cover some experimental, high cost” products, then any drugs that are left off are going to be ones that “have failed to meet reasonable standards of efficacy and/or cost-effectiveness.”^2^

Nearly all of the opponents of pharmacare talk about the cost of such a program. The Canadian Life and Health Insurance Association, in its brief to the federally appointed Advisory Council on the Implementation of National Pharmacare, said that expanding the current private/public system was the best way “to minimize the overall fiscal impact to government” while increasing coverage.^20^ As another example, the chief economist for the Canadian Chamber of Commerce said, “A single-payer plan will likely result in increased deficits and taxes, both of which are not in the interest of average Canadians.”^12^ This type of statement draws on the $19.3 billion estimated cost for pharmacare from the Parliamentary Budget Office.^33^ But that estimate assumes that the entire cost will be borne by the federal government, whereas most advocates for pharmacare are talking about a joint federal-provincial/territorial plan where the cost is shared by different levels of government. The Parliamentary Budget Office is also using as a model the Quebec formulary which is the most generous one in Canada^34^ and not necessarily the one that will form the basis for a national formulary.

What these naysayers want is for Canada to continue with its mix of public and private (insurance and out-of-pocket) payment that leaves the country with an annual per capita drug expenditure of US$806 against an OECD average of US$564. Public spending on prescription drugs in Canada is near the bottom of the OECD countries. Is it any wonder that we spend more per person than nearly all of them.^35^ One of the main reasons why other countries can keep their spending so much lower than Canada’s is because national drug insurance allows for monopsony buying power. How do these countries manage to afford national drug insurance without bankrupting themselves, is a question that opponents never address.

Drawing on an analogy from Evans and colleagues, the ideas of those opposed to pharmacare are like zombies.^6^ You think that you’ve killed them with rational arguments but they keep rising from the dead. It is time to deliver the final death blow and get on with delivering pharmacare and helping to complete the vision for a national public healthcare system for Canada.

## Ethical issues

Not applicable.

## Competing interests

In 2016-2019, JL was a paid consultant on two projects: one looking at developing principles for conservative diagnosis (Gordon and Betty Moore Foundation) and a second deciding what drugs should be provided free of charge by general practitioners (Government of Canada, Ontario Supporting Patient Oriented Research Support Unit and the St Michael’s Hospital Foundation). He also received payment for being on a panel at the American Diabetes Association, for a talks at the Toronto Reference Library, for writing a brief in an action for side effects of a drug for Michael F. Smith, Lawyer and a second brief on the role of promotion in generating prescriptions for Goodmans LLP and from the Canadian Institutes of Health Research for presenting at a workshop on conflict-of-interest in clinical practice guidelines. He is currently a member of research groups that are receiving money from the Canadian Institutes of Health Research and the Australian National Health and Medical Research Council. He is member of the Foundation Board of Health Action International and the Board of Canadian Doctors for Medicare. He receives royalties from University of Toronto Press and James Lorimer & Co. Ltd. for books he has written.

## Author’s contribution

JL is the single author of the paper.
